# Development and preliminary evaluation of a multiplexed amplification and next generation sequencing method for viral hemorrhagic fever diagnostics

**DOI:** 10.1371/journal.pntd.0006075

**Published:** 2017-11-20

**Authors:** Annika Brinkmann, Koray Ergünay, Aleksandar Radonić, Zeliha Kocak Tufan, Cristina Domingo, Andreas Nitsche

**Affiliations:** 1 Highly Pathogenic Viruses, ZBS 1, Centre for Biological Threats and Special Pathogens, Robert Koch Institute, Berlin, Germany; 2 Virology Unit, Department of Medical Microbiology, Faculty of Medicine, Hacettepe University, Ankara, Turkey; 3 Department of Infectious Diseases and Clinical Microbiology, Yıldırım Beyazıt University, Ankara, Turkey; Molecular Biology Unit (MBU), INDIA

## Abstract

**Background:**

We describe the development and evaluation of a novel method for targeted amplification and Next Generation Sequencing (NGS)-based identification of viral hemorrhagic fever (VHF) agents and assess the feasibility of this approach in diagnostics.

**Methodology:**

An ultrahigh-multiplex panel was designed with primers to amplify all known variants of VHF-associated viruses and relevant controls. The performance of the panel was evaluated via serially quantified nucleic acids from Yellow fever virus, Rift Valley fever virus, Crimean-Congo hemorrhagic fever (CCHF) virus, Ebola virus, Junin virus and Chikungunya virus in a semiconductor-based sequencing platform. A comparison of direct NGS and targeted amplification-NGS was performed. The panel was further tested via a real-time nanopore sequencing-based platform, using clinical specimens from CCHF patients.

**Principal findings:**

The multiplex primer panel comprises two pools of 285 and 256 primer pairs for the identification of 46 virus species causing hemorrhagic fevers, encompassing 6,130 genetic variants of the strains involved. In silico validation revealed that the panel detected over 97% of all known genetic variants of the targeted virus species. High levels of specificity and sensitivity were observed for the tested virus strains. Targeted amplification ensured viral read detection in specimens with the lowest virus concentration (1–10 genome equivalents) and enabled significant increases in specific reads over background for all viruses investigated. In clinical specimens, the panel enabled detection of the causative agent and its characterization within 10 minutes of sequencing, with sample-to-result time of less than 3.5 hours.

**Conclusions:**

Virus enrichment via targeted amplification followed by NGS is an applicable strategy for the diagnosis of VHFs which can be adapted for high-throughput or nanopore sequencing platforms and employed for surveillance or outbreak monitoring.

## Author summary

Viral hemorrhagic fever is a severe and potentially lethal disease, characterized by fever, malaise, vomiting, mucosal and gastrointestinal bleeding, and hypotension, in which multiple organ systems are affected. Due to modern transportation and global trade, outbreaks of viral hemorrhagic fevers have the potential to spread rapidly and affect a significant number of susceptible individuals. Thus, urgent and robust diagnostics with an identification of the causative virus is crucial. However, this is challenged by the number and diversity of the viruses associated with hemorrhagic fever. Several viruses classified in *Arenaviridae*, *Filoviridae*, and *Flaviviridae* families and *Bunyavirales* order may cause symptoms of febrile disease with hemorrhagic symptoms. We have developed and evaluated a novel method that can potentially identify all viruses and their genomic variants known to cause hemorrhagic fever in humans. The method relies on selected amplification of the target viral nucleic acids and subsequent high throughput sequencing technology for strain identification. Computer-based evaluations have revealed very high sensitivity and specificity, provided that the primer design is kept updated. Laboratory tests using several standard hemorrhagic virus strains and patient specimens have demonstrated excellent suitability of the assay in various sequencing platforms, which can achieve a definitive diagnosis in less than 3.5 hours.

## Introduction

Outbreaks of viral hemorrhagic fever (VHF) occur in many parts of the world [1,2]. VHFs are caused by various single-stranded RNA viruses, the majority of which are classified in *Arenaviridae*, *Filoviridae*, and *Flaviviridae* families and *Bunyavirales* order [3]. Human infections show high morbidity and mortality rates, can spread easily, and require rapid responses based on comprehensive pathogen identification [1,3,4]. However, routine diagnostic approaches are challenged when fast and simultaneous screening for different viral pathogens in higher numbers of individuals is necessary [5]. Even PCR as a widely used diagnostic method, usually providing specific virus identification, requires intense hands-on time for parallel screening of larger quantity of specimens and provides limited genetic information about the target virus. Multiplexing of different specific PCR assays aims at dealing with these drawbacks; however, until recently, it was limited to a few primer pairs in one reaction due to a lack of amplicon identification approaches for more than five targets [6,7].

Next Generation Sequencing (NGS) has provided novel options for the identification of viruses, including simultaneous and unbiased screening for different pathogens and multiplexing of various samples in a single sequencing run [8]. Furthermore, the development of real-time sequencing platforms has enabled processing and analysis of individual specimens within reasonable timeframes [9]. However, virus identification with NGS is also accompanied by major drawbacks, such as diminished sensitivity when viral genome numbers in the sample are insufficient and masked by unbiased sequencing of all nucleic acids present in the specimen, including the host genome [10,11]. Attempts to increase the sensitivity of NGS-based diagnostics have focused on enrichment of virus material and libraries before sequencing, including amplicon sequencing, PCR-generated baits, and solution-based capture techniques [12–14]. The strategy of ultrahigh-multiplex PCR with subsequent NGS has previously been employed for human single nucleotide polymorphism typing, genetic variations in human cardiomyopathies, and bacterial biothreat agents [15–17]. In this study, we describe the development and initial evaluation of a novel method for targeted amplification and NGS-identification of viral febrile disease and hemorrhagic fever agents and assess the feasibility of this approach in diagnostics.

## Methods

### Ethics statement

The human specimens, used for the evaluation of the developed panel were obtained from adults after written informed consent and in full compliance of the local ethics board approval (Ankara Research and Training Hospital, 13.07.11/0426).

### Panel design

Viruses reported to cause VHF as well as related strains, associated with febrile disease accompanied by arthritis, respiratory symptoms, or meningoencephalitis, were included in the design to enable differential diagnosis (Table 1). For each virus strain, all genetic variants with complete or near-complete genomes deposited in GenBank (https://www.ncbi.nlm.nih.gov/genbank/) were assembled into groups of >90% nucleotide sequence identity via the Geneious software (version 9.1.3) [18]. The consensus sequence of each group was included in the design. The primer sequences were deduced using the Ion AmpliSeq Designer online tool (https://ampliseq.com/browse.action) which provides a custom multiplex primer pool design for NGS (Thermo Fisher Scientific, Waltham, MA). For initial evaluation of the approach and as internal controls, human-pathogenic viruses belonging in identical and/or distinct families/genera but not associated with hemorrhagic fever or febrile disease were included in the design (Table 1).

**Table 1:** List of the viruses targeted by the ultrahigh-multiplex assay.

Family	Genus	Strain / Species
*Arenaviridae*	*Mammarenavirus*	Chapare mammarenavirus*
		Guanarito mammarenavirus*
		Junin mammarenavirus*
		Lassa mammarenavirus*
		Lujo mammarenavirus*
		Machupo mammarenavirus*
		Pichinde mammarenavirus*
		Sabia mammarenavirus*
*Nairoviridae*	*Orthonairovirus*	Crimean-Congo Hemorrhagic fever virus*
		Dugbe virus
*Peribunyaviridae*	*Orthobunyavirus*	Oropouche virus
*Hantaviridae*	*Orthohantavirus*	Dobrava-Belgrade virus*
		Hantaan virus*
		New York hantavirus
		Puumala virus*
		Saaremaa virus*
		Seoul virus*
		Tula virus
*Phenuiviridae*	*Phlebovirus*	Heartland virus
		Sandfly fever Sicilian virus
		Severe fever with thrombocytopenia syndrome virus*
		Punta Toro virus
		Rift Valley fever virus*
		Toscana virus
		Uukuniemi virus
*Filoviridae*	*Cuevavirus*	Lloviu cuevavirus*
	*Ebolavirus*	Bundibugyo virus*
		Reston ebolavirus*
		Sudan ebolavirus*
		Tai Forest ebolavirus*
		Zaire ebolavirus*
	*Marburgvirus*	Marburg marburgvirus*
*Flaviviridae*	*Flavivirus*	Yellow fever virus*
		Dengue virus 1*
		Dengue virus 2*
		Dengue virus 3*
		Dengue virus 4*
		Kyasanur Forest disease virus*
		Omsk hemorrhagic fever virus*
		West Nile virus
	*Hepavivirus*	Hepatitis C virus
*Hepadnaviridae*	*Orthohepadnavirus*	Hepatitis B virus
*Hepeviridae*	*Orthohepevirus*	Hepatitis E virus
*Picornaviridae*	*Hepatovirus*	Hepatitis A virus
*Rhabdoviridae*	*Tibrovirus*	Bas-Congo virus*
*Togaviridae*	*Alphavirus*	Barmah Forest virus
		Chikungunya virus
		Mayaro virus
		O'nyong-nyong virus
		Sindbis virus
*-*	*Deltaviridae*	Hepatitis D virus

* documented as a confirmed or probable causative agent of VHF in humans

### *In silico* panel evaluation

The designed primers were tested *in silico* for specific binding to the target virus strains, including all known genotypes and genetic variants. The primer sets were aligned to their specific target reference sequences and relative primer orientation, amplicon size and overlap, and total mismatches for each primer were evaluated using the Geneious software [18]. Pairs targeting a specific virus with less than two mismatches in sense and antisense primers were defined as a hit and employed for sensitivity calculations. Unspecific binding of each primer to non-viral targets was investigated via the BLASTn algorithm, implemented within the National Center for Biotechnology Information website (https://blast.ncbi.nlm.nih.gov/Blast.cgi) [19]. The sensitivity and specificity of the primer panel for each virus were determined via standard methods as described previously [20].

### Panel evaluation via virus strains

The performance of the novel panel for the detection of major VHF agents was evaluated via selected virus strains. For this purpose, nucleic acids from Yellow fever virus (YFV) strain 17D, Rift Valley fever virus (RVFV) strain MP-12, Crimean-Congo hemorrhagic fever virus (CCHFV) strain UCCR4401, Zaire Ebola virus (EBOV) strain Makona-G367, Chikungunya virus (CHIKV) strain LR2006-OPY1 and Junin mammarenavirus (JUNV) strain P3766 were extracted with the QIAamp Viral RNA Mini Kit (Qiagen, Hilden, Germany) with subsequent cDNA synthesis according to the SuperScript IV Reverse Transcriptase protocol (Thermo Fisher Scientific). Genome concentration of all strains was determined by specific quantitative real-time PCRs using plasmid-derived virus standards, as described previously (protocols are available upon request). Genome equivalents (ge) of 10^0^–10^3^ for each virus were prepared and mixed with 10 ng of human genetic material recovered from HeLa cells.

In order to compare the efficiency of amplification with the novel panel versus direct NGS, all virus cDNAs were further subjected to second strand cDNA synthesis using the NEBNext RNA Second Strand Synthesis Module (New England BioLabs GmbH, Frankfurt, Germany) according to the manufacturer’s instructions. Reagent-only mixes and HeLa cell extracts were employed as negative controls in the experiments.

### Panel evaluation via human specimens

The performance of the panel was further tested on clinical specimens from individuals with a clinical and laboratory diagnosis of VHF [21]. For this purpose, previously stored sera with quantifiable CCHFV RNA and lacking IgM or IgG antibodies were employed and processed via High Pure Viral Nucleic Acid Kit (Roche, Mannheim, Germany) and the SuperScript IV Reverse Transcriptase (Thermo Fisher Scientific) protocols, as suggested by the manufacturer. Two human sera, without detectable nucleic acids of the targeted viral strains were tested in parallel as negative controls.

### PCR amplification

The specimens were amplified using the custom primer panels designed for HFVs with the following PCR conditions for each pool: 2 μl of viral cDNA mixed with human genetic material, 5 μl of primer pool, 0.5 mM dNTP (Invitrogen, Karlsruhe, Germany), 5 μl of 10 x Platinum Taq buffer, 4 mM MgCl_2_, and 10 U Platinum Taq polymerase (Invitrogen) with added water to a final volume of 25 μl. Cycling conditions were 94°C for 7 minutes, 45 amplification cycles at 94°C for 20 seconds, 60°C for 1 minute, and 72°C for 20 seconds, and a final extension step for 6 minutes (at 72°C). Thermal cycling was performed in an Eppendorf Mastercycler Pro (Eppendorf Vertrieb Deutschland, Wesseling-Berzdorf, Germany) with a total runtime of 90 minutes.

### Library preparation and NGS sequencing

The amplicons obtained from the virus strains were subjected to the Ion Torrent Personal Genome Machine (PGM) System for NGS analysis (Thermo Fisher Scientific Inc.). Initially, the specimens were purified with an equal volume of Agencourt AMPure XP Reagent (Beckman Coulter, Krefeld, Germany). PGM libraries were prepared according to the Ion Xpress Plus gDNA Fragment Library Kit, using the “Amplicon Libraries without Fragmentation” protocol (Thermo Fisher Scientific). For direct NGS, specimens were fragmented with the Ion Shear Plus Reagents Kit (Thermo Fisher Scientific) with a reaction time of 8 minutes. Subsequently, libraries were prepared using the Ion Xpress Plus gDNA Fragment Library Preparation kit and associated protocol (Thermo Fisher Scientific). All libraries were quality checked using the Agilent Bioanalyzer (Agilent Technologies, Frankfurt, Germany), quantitated with the Ion Library Quantitation Kit (Thermo Fisher Scientific), and pooled equimolarly. Enriched, template-positive Ion PGM Hi-Q Ion Sphere Particles were prepared using the Ion PGM Hi-Q Template protocol with the Ion PGM Hi-Q OT2 400 Kit (Thermo Fisher Scientific). Sequencing was performed with the Ion PGM Hi-Q Sequencing protocol, using a 318 chip.

Amplicons obtained from CCHFV-infected individuals and controls were processed for nanopore sequencing via MinION (Oxford Nanopore Technologies, Oxford, United Kingdom). The libraries were prepared using the ligation sequencing kit 1D, SQK-LSK108, R9.4 (Oxford Nanopore Technologies). Subsequently, the libraries were loaded on Oxford Nanopore MinION SpotON Flow Cells Mk I, R9.4 (Oxford Nanopore Technologies) using the library loading beads and run until initial viral reads were detected.

### Bioinformatic analysis

The sequences generated by PGM sequencing were trimmed to remove adaptors from each end using Trimmomatic [22], and reads shorter than 50 base pairs were discarded. All remaining reads were mapped against the viral reference database prepared during the design process via Geneious 9.1.3 software [18]. During and after MinION sequencing, all basecalled reads in fast5 format were extracted in fasta format using Poretools software [23]. The BLASTn algorithm was employed for sequence similarity searches in the public databases when required.

## Results

### The VHF panel and *in silico* performance

The AmpliSeq design for the custom multiplex primer panel resulted in two pools of 285 and 256 primer pairs for the identification of 46 virus species causing hemorrhagic fevers, encompassing 6,130 genetic variants of the strains involved. All amplicons were designed to be within a range of 125–375 base pairs. Melting temperature values of the primers ranged from 55.3°C to 65.0°C. No amplicons <1,000 base pairs with primer pairs in relative orientation and distance to each other could be identified, leading to an overall specificity of 100% for all virus species. The primer sequences in the panels are provided in S1 Table.

**Fig 1:**
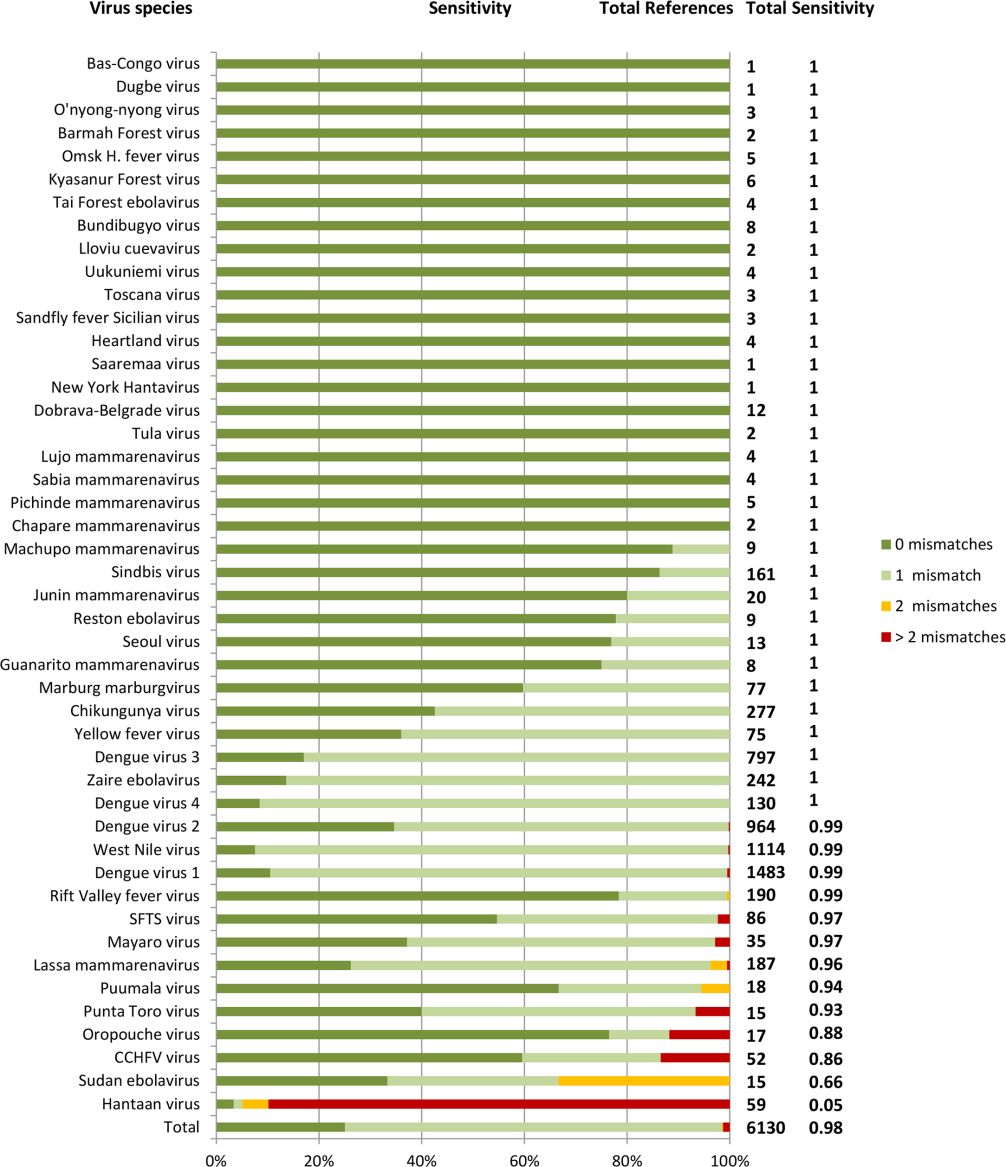
Sensitivity of the designed primers according to the target virus genetic variants referenced in GenBank. Total number of reference sequences and PCR sensitivity for each virus are indicated on the y-axis. Numbers of mismatches in the primers are encoded with colours.

The overall sensitivity of the panel reached 97.9%, with the primer pairs targeting 6,007 out of 6,130 genetic variants (1 mismatch in one or both of each primers of a primer pair accepted, as described above) (Fig 1). Impaired sensitivity was noted for Hantaan virus (0.05). Evaluation of all Hantaan virus variants in GenBank revealed that newly added virus sequences were divergent by up to 17% from sequences included in the panel design, leading to diminished primer binding. These sequences could be fully covered by two sets of additional primers.

### Comparison of direct and targeted NGS

**Fig 2:**
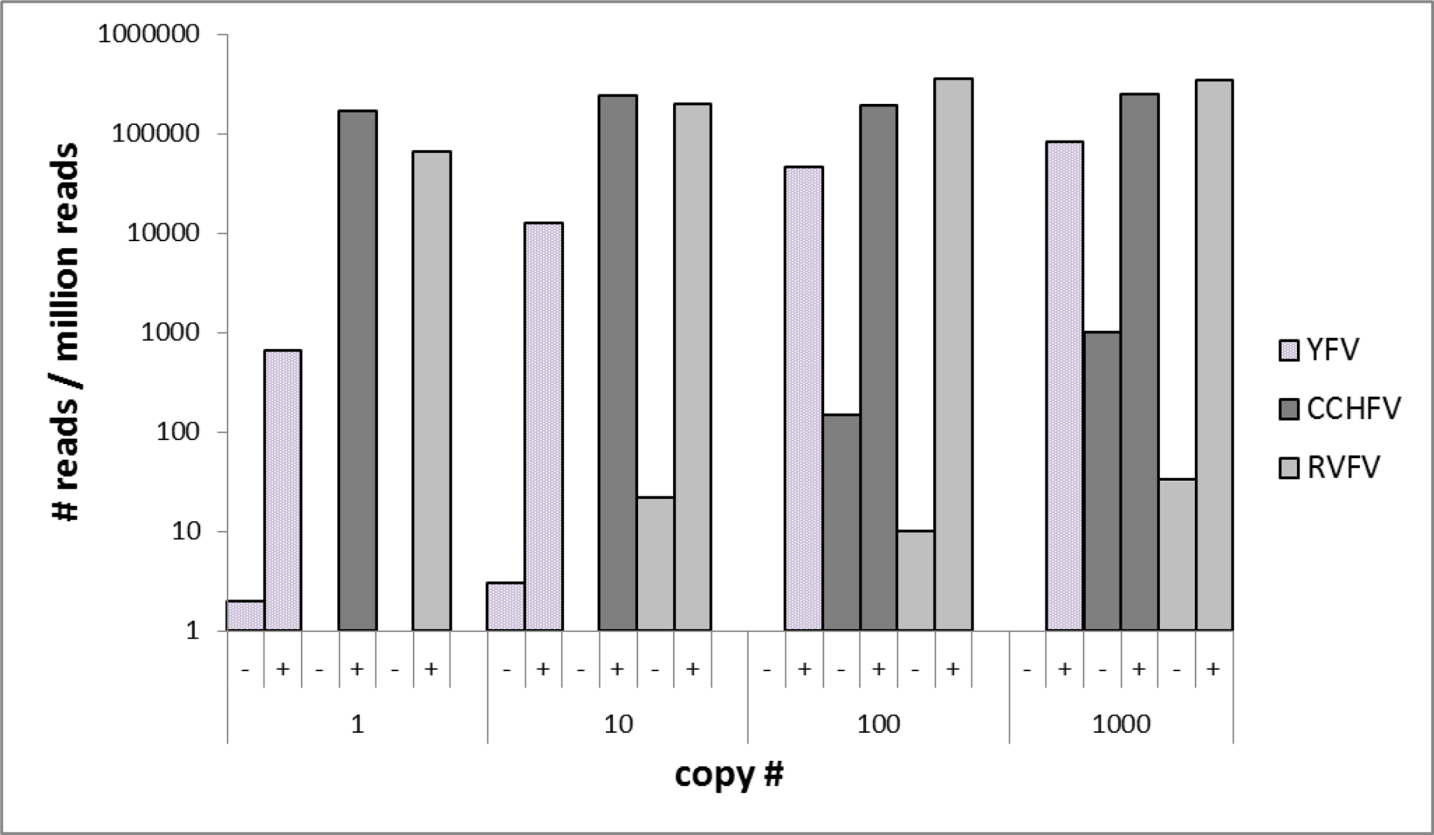
Number of virus-specific amplicons detected via semiconductor sequencing in 1 million total reads with (+) or without (-) amplification with the novel panel (YFV: Yellow fever virus; RVFV: Rift Valley fever virus; CCHFV: Crimean-Congo hemorrhagic fever virus).

**Fig 3:**
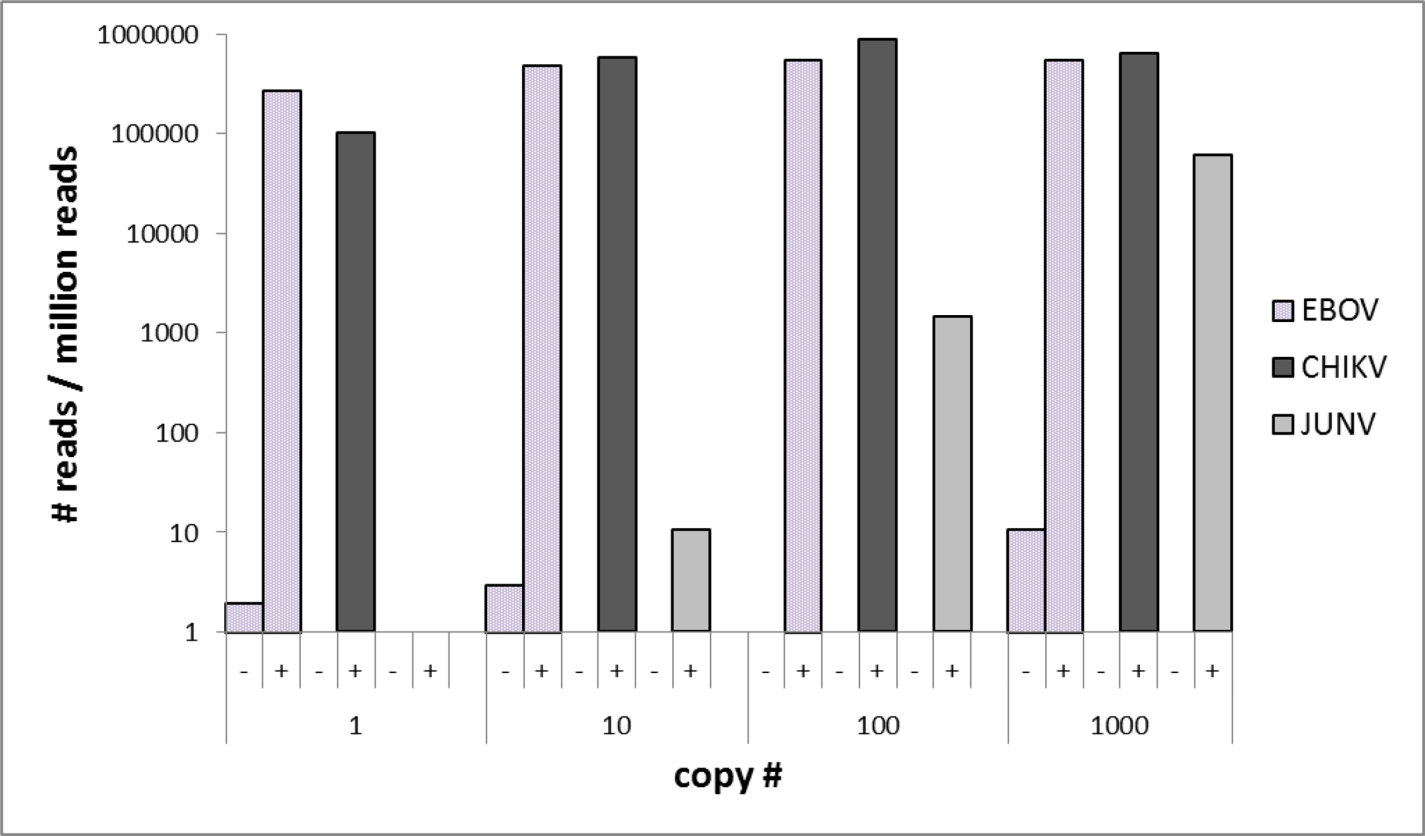
Number of virus-specific amplicons detected via semiconductor sequencing in 1 million total reads with (+) or without (-) amplification with the novel panel (EBOV: Ebola virus; CHIKV: Chikungunya virus; JUNV: Junin virus).

Amplification of viral targets with the multiplex PCR panel prior to NGS resulted in a significant increase of viral read numbers compared to direct NGS (Figs 2 and 3, S2 Table). In specimens with 10^3^ ge of the target strain, the ratio of viral reads to unspecific background increased from 1×10^−3^ to 0.25 (CCHFV), 3×10^−5^ to 0.34 (RVFV), 1×10^−4^ to 0.27 (EBOV), and 2×10^−5^ to 0.64 (CHIKV) with fold-changes of 247, 10,297, 1,633, and 25,398, respectively. In direct NGS, no viral reads could be detected for CCHFV and CHIKV genomic concentrations lower than 10^3^, and this approach failed to identify YFV and JUNV regardless of the initial virus count. In targeted NGS, the limit of detection was noted as 10^0^ ge for YFV, CCHFV, RVFV, EBOV, and CHIKV and 10^1^ ge for JUNV. For the viruses detectable via direct NGS, amplification provided significant increases in specific viral reads over total reads ratios, from 10^−4^ to 0.19 (CCHFV, 1,900-fold change), 2×10^−5^ to 0.19 (RVFV, 9,500-fold change), and 3×10^−4^ to 0.56 (EBOV, 1,866-fold change). The average duration of the workflow of direct and targeted NGS via PGM was 19 and 20.5 hours, respectively.

### Patient specimen findings

In all patient sera evaluated via nanopore sequencing following amplification, the causative agent could be detected after 1 to 9 minutes of the NGS run (Table 2). The characterized sequences were 89–99% identical to the CCHFV strain Kelkit L segment (GenBank accession: GQ337055) known to be in circulation in Turkey [24, 25]. No targeted viral sequence could be observed in human sera used as negative controls during 1 hour of sequencing. The preparation, amplification, and sequencing steps of the clinical specimens could be completed with a total sample-to-result time of less than 3.5 hours.

**Table 2:** Findings of the targeted amplification via the novel panel and real-time nanopore sequencing in patient serum specimens with CCHFV-induced hemorrhagic fever.

#	Origin	Virus identified	Virus load^1^	Viral reads	Total reads	Time to first viral read	Processing time^2^
**1**	Turkey, Ankara	CCHFV-Kelkit06	1.96 x 10^6^	39	3860	9 min	189 min
**2**	Turkey, Ankara	CCHFV-Kelkit06	5.20 x 10^7^	4	1244	1 min	181 min
**3**	Turkey, Ankara	CCHFV-Kelkit06	1.90 x 10^6^	97	8025	1 min	181 min

^1^ (ge/ml)

^2^ includes all specimen processing steps until definitive diagnosis.

## Discussion

In this study, we report the development and evaluation of an ultrahigh-multiplex PCR for the enrichment of viral targets before NGS, which aims to provide a robust molecular diagnosis in VHFs. The panel was observed to be highly specific and sensitive and to have the capacity to detect over 97% of all known genetic variants of the targeted 46 viral species *in silico*. The sensitivity of the primer panel was impaired by virus sequences not included in the original design, as noted for Hantaan virus in this study. As 36 out of a total of 59 isolates have been published after panel design was completed, these genetic variants of Hantaan virus could not be detected with a comparable sensitivity or not at all with the current panel. This indicates that the panel has to be adapted to newly-available sequences in public databases. We have evaluated how the panel could be updated to accommodate these recently-added sequences and observed that two additional primer pairs could sufficiently cover all divergent entries. Although the approach for the panel design as well as the actual design with the AmpliSeq pipeline was successful for all genetic variants included, the amplification of viral sequences significantly diverging from the panel could not be guaranteed, which may also apply for novel viruses. Unlike other pathogenic microorganisms, viruses can be highly variable in their genome. Only rarely do they share genes among all viruses or virus species that could be targeted as a virus-generic marker by amplification. Our strategy for primer design and the AmpliSeq pipeline do not permit the generation of degenerated primers or the targeting of very specific consensus sequences. However, the design of the primer panel is relatively flexible, and additional primer pairs can be appended in response to recently published virus genomes. Moreover, an updated panel will also encompass non-viral pathogens relevant for differential diagnosis, and syndrome-specific panels targeting only VHF agents or virally induced febrile diseases such as West Nile fever and Chikungunya can be developed.

We have further tested the panel using quantitated nucleic acids of six well-characterized viruses responsible for VHF or severe febrile disease, with a background of human genetic material to simulate specimens likely to be submitted for diagnosis, using the semiconductor PGM sequencing platform. The impact of amplification was evaluated with a comparison of direct and amplicon-based NGS runs. Overall, targeted amplification prior to NGS ensured viral read detection in specimens with the lowest virus concentration (1 ge) in five of the six viruses evaluated and 10 ge in the remaining strain, which is within the range of the established real-time PCR assays. Furthermore, this approach enabled significant increases in specific viral reads over background in all of the viruses, with varying fold changes in different strains and concentrations (Figs 2 and 3). The increased sensitivity and specificity provided with the targeted amplification suggest that it can be directly employed for the investigation of suspected VHF cases where viremia is usually short and the time point of maximum virus load is often missed [1,5].

Finally, we evaluated the VHF panel by using serum specimens obtained during the acute phase of CCHFV-induced disease and employed an alternate NGS platform based on nanopore sequencing. This approach enabled virus detection and characterization within 10 minutes of the NGS run and can be completed in less than 3.5 hours in total (Table 2). The impact of the nanopore sequencing has been revealed previously, during the EBOV outbreak in West Africa where the system provided an efficient method for real-time genomic surveillance of the causative agent in a resource-limited setting [26]. Field-forward protocols based on nanopore sequencing have also been developed recently for pathogen screening in arthropods [27]. Specimen processing time is likely to be further reduced via the recently developed rapid library preparation options. While the duration of the workflow is longer, the PGM and similar platforms are well-suited for the parallel investigation of higher specimen numbers.

Although we have demonstrated in this study that targeted amplification and NGS-based characterization of VHF and febrile disease agents is an applicable strategy for diagnosis and surveillance, there are also limitations of this approach. In addition to the requirement of primer sequence updates, the majority of the workflow requires non-standard equipment and well-trained personnel, usually out of reach for the majority of laboratories in underprivileged geographical regions mainly affected by these diseases. However, NGS technologies are becoming widely available with reduced total costs and can be swiftly transported and set up in temporary facilities in field conditions [26,27]. During outbreak investigations, where it is impractical and expensive to test for several individual agents via specific PCRs, this approach can easily provide information on the causative agent, facilitating timely implementation of containment and control measures. Additional validation of the approach will be provided with the evaluation of well-characterized clinical specimen panels and direct comparisons with established diagnostic assays.

In conclusion, virus enrichment via targeted amplification followed by NGS is an applicable method for the diagnosis of VHFs which can be adapted for high-throughput or nanopore sequencing platforms and employed for surveillance or outbreak monitoring.

## Supporting information

S1 Table Sequence of the primers included in the custom multiplex pool. (XLS)

S2 Table Viral and total read numbers obtained via direct and amplified NGS for the VHF agents tested. (XLS)
